# Presence of Antigen-Experienced T Cells with Low Grade of Differentiation and Proliferative Potential in Chronic Chagas Disease Myocarditis

**DOI:** 10.1371/journal.pntd.0002989

**Published:** 2014-08-21

**Authors:** Rafael J. Argüello, Carlos Vigliano, Patricia Cabeza-Meckert, Rodolfo Viotti, Fernando Garelli, Liliana E. Favaloro, Roberto R. Favaloro, Rubén Laguens, Susana A. Laucella

**Affiliations:** 1 Instituto Nacional de Parasitología “Dr. Mario Fatala Chabén”, Buenos Aires, Argentina; 2 Departamento de Patología, Hospital Universitario Fundación Favaloro, Buenos Aires, Argentina; 3 Servicio de Cardiología, Sección de Chagas, Hospital Interzonal General de Agudos “Eva Perón”, San Martín, Buenos Aires, Argentina; 4 Laboratorio de Eco-Epidemiología de la enfermedad de Chagas, Facultad de Ciencias Exactas y Naturales, Universidad de Buenos Aires, Buenos Aires, Argentina; 5 Departamento de Trasplante Intratorácico. Hospital Universitario Fundación Favaloro, Buenos Aires, Argentina; Federal University of São Paulo, Brazil

## Abstract

**Background:**

The main consequence of chronic *Trypanosoma cruzi* infection is the development of myocarditis in approximately 20–30% of infected individuals but not until 10–20 years after the initial infection. We have previously shown that circulating interferon-γ-secreting T cells responsive to *Trypanosoma cruzi* antigens in chronic Chagas disease patients display a low grade of differentiation and the frequency of these T lymphocytes decreases along with the severity of heart disease. This study thought to explore the expression of inhibitory receptors, transcription factors of type 1 or regulatory T cells, and markers of T cell differentiation, immunosenescence or active cell cycle in cardiac explants from patients with advanced Chagas disease myocarditis.

**Methodology/Principal Findings:**

The expression of different markers for T and B cells as well as for macrophages was evaluated by immunohistochemistry and immunofluorescence techniques in cardiac explants from patients with advanced chronic Chagas disease submitted to heart transplantation. Most infiltrating cells displayed markers of antigen-experienced T cells (CD3^+^, CD4^+^, CD8^+^, CD45RO^+^) with a low grade of differentiation (CD27^+^, CD57^−^, CD45RA^−^, PD-1^−^). A skewed T helper1/T cytotoxic 1 profile was supported by the expression of T-bet; whereas FOXP3^+^ cells were scarce and located only in areas of severe myocarditis. In addition, a significant proliferative capacity of CD3^+^ T cells, assessed by Ki67 staining, was found.

**Conclusions/Significance:**

The quality of T cell responses and immunoregulatory mechanisms might determine the pattern of the cellular response and the severity of disease in chronic *Trypanosoma cruzi* infection.

## Author Summary

Chagas disease is a neglected tropical disease affecting approximately 10 million people in the world. It is caused by infection with the protozoan *Trypanosoma cruzi*. As a consequence of migration flows, the disease has been also become established in non-endemic countries. In this study, the functional and phenotypic profile of inflammatory T cells were evaluated in heart tissues of patients with end-stage chronic Chagas disease by analyzing the expression of inhibitory receptors, transcription factors of type 1 or regulatory T cells, and markers of T cell differentiation, immunosenescence or active cell cycle. Most infiltrating cells displayed markers of antigen-experienced T cells with a low grade of differentiation and a significant proliferative capacity. A skewed T helper1/T cytotoxic 1 profile was supported by the expression of T-bet; whereas FOXP3^+^ cells were scarce. The quality of T cell responses and immunoregulatory mechanisms might determine the pattern of the cellular response and the severity of disease in *Trypanosoma cruzi* infection.

## Introduction

The main consequence of chronic *Trypanosoma cruzi (T. cruzi)* infection is the development of myocarditis in approximately 20–30% of infected individuals but not until 10–20 years after the initial infection [1]. Advanced chronic Chagas heart disease (cChHD) is characterized by dilated cavities with high degree of fibrosis and inflammation [2], [3]. The analysis by immunohistochemical, but mainly by molecular techniques, of cardiac samples from chronically *T. cruzi*-infected subjects provided evidence of the association between parasite persistence and tissue damage in cChHD [2], [4], [5]. Reis et al. showed that inflammatory lesions were dominated by CD8^+^ lymphocytes, many of which expressed granzyme A [6]. Lymphocytes in these lesions express lymphocyte function antigen-a (LFA-1), CD44, very late antigen-4 (VLA-4) [7] and cytotoxic lymphocyte antigen 4 (CTLA-4) [8]. A Th1 cytokine pattern predominated in the cardiac inflammatory cell infiltrate of Chagas disease patients with heart failure [9].Whereas some authors have shown increased peripheral levels of IFN-γ in patients with severe heart disease [10]–[12], other studies have demonstrated an inverse association between disease severity and IFN-γ production [8], [13], [14].We have previously shown that most IFN-γ-secreting T cells in response to *T. cruzi* display a low grade of differentiation but high expression of the inhibitory receptor CTLA-4 in the circulation of subjects with chronic *T. cruzi* infection [8], [15], [16]. Conversely, the total T cell compartment in Chagas disease patients is enriched in highly differentiated T cells compared to uninfected controls [15]–[17].

There is very limited data available on the degree of differentiation of T cells in heart lesions of cChHD, and a comprehensive analysis of the relationship of different T cell functions in Chagas disease myocarditis has not been performed. This study thought to explore the expression of inhibitory receptors, transcription factors of type 1 or regulatory T cells and markers of T cell differentiation, immunosenescence or active cell cycle in cardiac explants from patients with advanced cChHD submitted to heart transplantation.

## Materials and Methods

### Ethics statement

This study was approved by the Institutional Review Boards of the Hospital Universitario Fundación Favaloro (UIC (863) 1604), and all patients gave written informed consent for the heart transplant procedure.

### Patients

Eight patients with severe cChHD (4 men and 4 women; mean age ± SD, 51.4±7.3 years) were admitted at Hospital Universitario Fundación Favaloro in Buenos Aires, Argentina, during the period 1998–2008 to undergo orthotopic heart transplantation. Diagnosis of *T. cruzi* infection was confirmed when at least 2 out of 3 standard serological tests (enzyme-linked immunosorbent assay, indirect hemagglutination and immunofluorescence).were positive. Determination of cChHD was based on clinical, echocardiographic and electrocardiographic findings. Explanted hearts from patients with Giant cell myocarditis (GCM; n = 2) and idiopathic dilated cardiomyopathy (IDCM; n = 1) were also included as controls. Human lymph node and placental tissues from the Tissue Bank of the Pathology Lab were employed as positive staining controls. The cChHD patients included in this study had not received immunosuppressive drugs, trypanocidal therapy or prophylactic benznidazole by the time of this study.

### Analysis of heart explants

Eight explanted hearts were weighed and fixed for 72 h in 10% phosphate-buffered formaldehyde. After fixation, transmural sections of the whole circumference of the left and right ventricle at a plane equidistant from the base to the apex were collected and embedded in paraffin. A 5-mm-thick section from each region was stained with hematoxylin and eosin and Masson's trichrome solution. The interventricular septum of each heart was selected for histological and immunohistochemistry analysis. The diagnosis of myocarditis was defined according to the Dallas criteria taking into account the inflammatory infiltrate of the myocardium and the presence of necrosis and/or degeneration of adjacent myocytes [18]. The distribution of the inflammatory infiltrate was classified as focal, confluent or diffuse [19].

The median number of lymphocytes plus macrophages identified by the expression of CD3 and CD68, respectively, was calculated for 8 tissue samples from cChHD, 2 GCM samples and 1 IDCM sample assessed, as described in “Quantification of cells” [20]. Myocarditis recorded in each tissue sample was considered as severe when the number of lymphocytes plus macrophages was over the median number of these cell types in cChHD, moderate when the number of lymphocytes plus macrophages was between 25^th^ and 50^th^ percentile, and mild when the number of lymphocytes plus macrophages was under percentile 25^th^.

For quantitative assessment of fibrosis an interventricular septum block that was embedded in paraffin and sectioned at 5 µm was stained with picrosirius red. After obtaining digital images with a digital scanner (UMAX Technologies Inc., USA) at 2× magnification and a 1200 ppp resolution, the percentage of the surface area occupied by collagen was established by morphometric analysis using the digital analysis system Image Pro Plus 4.5 (Media Cybernetics, Silver Spring, USA) [21]. The percentage of fibrosis was semi-quantified as mild (<10%), moderate (10%–20%), or severe (>20%) [22]. Molecular detection of *T. cruzi* in the same interventricular tissue sections by PCR was previously performed [23]. The presence of *T. cruzi* was also analyzed by direct observation of intracellular amastigotes.

### Immunohistochemistry

Formalin fixed paraffin embedded tissue sections were rehydrated. Heat induced antigen retrieval, incubation time and antibody concentrations were selected following the manufacturer's recommendations. For CD3 (Mouse monoclonal, Santa Cruz Biotechnology, USA), CD8 (Leica Microsystems, Germany), CD68 (Biogenex, USA), CD20 (Biogenex, USA), CD45RA (Biogenex, USA), CD21 (Biogenex, USA), PD-1 (Abcam plc, UK), CD27 (Leica Microsystems, Germany), Ki67 (Rabbit monoclonal from Abcam plc, UK, and mouse monoclonal HLA-G from Leica Microsystems, Germany) and T-bet (BD, Biosciences, USA), heat induced antigen retrieval was done using Antigen Retrieval Citra Plus (Citrate buffer buffer based Ag retrieval solution pH = 6; Biogenex, USA). Antigen retrieval solution EZ-AR2™ (EDTA based retrieval buffer pH = 9; Biogenex, USA) was used to detect CD4 (Leica Microsystems, Germany) and FOXP3 (BD, Biosciences, USA) expression. For CD57 (BD, Biosciences, USA) and CD45RO expression (Biogenex, USA), no antigen retrieval after rehydration was done. Biotinylated anti mouse immunoglobulin G, peroxidase labeled streptavidin, and AEC (3-amino-9-ethyl carbazole) as chromogen were used as secondary detection system (Biogenex, USA). All immunohistochemistry slides were counterstained with hematoxylin. The list of antibodies and clones used, as well the function of each marker are depicted in Table S1 [24]–[37].

### Immunofluorescence

Tissue sections with high inflammation from patients 1 to 4 were selected for double labeling studies by immunofluorescence. Double labeling assays were carried out by staining with a combination of anti-Ki67 (rabbit monoclonal antibody)/anti-CD8; anti-KI67/anti-CD20 (mouse mAbs), anti-Ki67 (mouse mAb)/anti-CD3 (rabbit polyclonal) and anti-Ki67/anti-CD21 (rabbit monoclonal) (Table 1). Fluorescein labeled anti-rabbit goat immunoglobulin (Vector, USA) and Alexa fluor 594 labeled anti-mouse immunoglobulin (Invitrogen, USA) were used as secondary detection system. Nuclei staining were done with ready to use mounting medium for fluorescence with DAPI (Vectashield, Vector, USA). Antibody dilutions were used according to the manufacturer's instructions. Observations were made with a 100 W ultraviolet lamp and photographed with an AXIOCAM camera (Carl Zeiss AG, Germany).

**Table 1:** Demographic, clinical and pathologic characteristics of study patients.

Case	Gender	Age	Etiology	NYHA FC*	LVEDD† (mm)	LVEF‡ (%)	Heart weight/body weight (g/Kg))	Myocarditis Median number of CD3^+^+CD68^+^ cells (IQR§)^A^	Fibrosis (%)^B^	Parasite nests^C^
1	M	42	cChHD§	IV	63	15	410/75	287 (144–394)	45	Yes
2	F	48	cChHD	IV	72	33	420/48.9	116 (79–185)	23	No
3	M	45	cChHD	III	75	13	495/61	117 (63–166)	11	No
4	M	46	cChHD	III	77	14	460/66.6	72 (42–83)	21	No
5	M	59	cChHD	III	65	21	460/54.5	20 (18–54)	12	No
6	F	51	cChHD	IV	47	26	555/71	33 (25–47)	7	No
7	F	61	cChHD	III	61	14	335/54.7	18 (13–26)	22	No
8	F	59	cChHD	IV	76	18	465/51.9	14 (8–26)	20	No
A	M	28	GCM¶	IV	59	25	395/87	217 (187–266)	31	No
B	M	47	GCM	IV	61	20	355/105	185 (101–243)	61	No
C	M	46	iDCM‖‖	IV	78	30	510/79.5	9 (4–12)	11	No

A The degree of myocarditis was determined according to the number of lymphocytes plus macrophages in cChHD: Severe >48 median CD3^+^+CD68^+^/HPF, Moderate 20–48 median CD3^+^+CD68^+^/HPF, Mild <20 median CD3^+^+CD68^+^/HPF.

B The percentage of fibrotic area was determined by morphometric analysis as described in Materials and Methods [21].

C Parasite DNA by PCR was detected in heart sections of all cChHD, except for patient 6 [23]. Abbreviations:

* NYHA FC, New York Heart Association, functional class;

† LVEDD, left ventricular end-diastolic diameter;

‡ LVEF, left ventricular ejection fraction;

§ cChHD, chronic Chagas heart disease;

¶ GCM, Giant Cell Myocarditis;

‖‖ iDCM, idiopathic dilated cardiomyopathy;

§ IQR, interquartile range; HPF, high power field.

### Quantification of cells

For quantification of the total number of mononuclear inflammatory cells (i.e. cells with positive and negative staining for each marker assessed), 10 High Power Field (HPF) at 400× were counted for each section (1 section for each one of the 13 markers assessed). The median number of mononuclear inflammatory cells in 130 HPF was calculated for each patient.

The total number of mononuclear inflammatory cells with positive staining for each marker assessed was counted in 10 HPF, Magnification 400×. The percentage of cells expressing each marker was calculated by the ratio between the median number of positive cells for each marker and the median number of total mononuclear cell count in 10 HPF.

Cell counting was manually implemented using the Cell Counter plug-in of Image J 1.45b software (National Institutes of Health, USA). The position of the cells in each HPF was determined by Cell Counter plug-in and utilized thereafter for spatial histological analysis. This analysis was carried out in each one of the eight samples from Chagas disease patients, as well as in control samples.

### Statistical analysis

Continuous variables are reported as means (SDs) or medians (interquartile range –IQR-), while categorical variables are presented as the percentage of subjects out of total subjects evaluated. Continuous variables with non-Gaussian distribution were analyzed by Mann-Whitney *U* test. Correlation analysis between the percentage of cells positive for each marker and the total number of mononuclear inflammatory cells was done using the Spearman correlation test.

In order to estimate the spatial pattern of infiltrating cells surrounding a *T. cruzi* –infected cardiomyocyte, a spatial point pattern analysis was applied. A focal analysis of the distribution of CD45RO^+^ cells around an infected cardiomyocyte was performed by the bivariate Wiegand-Moloney O-ring statistic [38]. This test allows the characterization of a spatial pattern around a point at varying distances to the point, detecting aggregation, repulsion or randomness. The analysis was implemented with software Programita, using a random labeling null model. Confidence envelopes were calculated using 999 Montecarlo simulations [39]. A p value<0.05 (2-tailed) was considered statistically significant. For statistical analysis SPSS 11.0 statistical software (SPSS Inc, USA) was used.

## Results

### Clinical and demographical characteristics of cChHD patients

The clinical and demographical characteristics of cChHD patients are depicted in Table 2. Patients 1–5 and 7–8 were in end-stage cChHD, whereas patient 6 displayed cChHD and concomitant valvular heart disease. This patient suffered from rheumatic fever during her childhood, but no evidence of active endocarditis was observed by the time of the study. All patients were in the New York Heart Association classes III and IV by the time of transplantation. Echocardiographic studies revealed moderate to severe dilation of the cavities with a mean left ventricular end-diastolic diameter of 67.0±10.2 mm. The mean left ventricular ejection fraction, determined by radionuclide ventriculography, was 19.2±7.1%.

**Table 2:** Phenotypic and functional profile of inflammatory cells in heart tissues from cChHD, GCM and IDCM.

Case	CD3	CD8	CD4	CD68	CD20	CD57	PD-1	CD45RO	CD27	Ki67	T-bet	FOXP3	HLA-G
1	203/407	122/306	104/397	65/403	12/177	3/279	1/291	172/282	74/241	40/350	80/296	0/180	0/408
	(49.9)	(39.9)	(26.2)	(16.1)	(6.8)	(1.1)	(0.3)	(61.0)	(30.7)	(11.4)	(27.0)	(0)	(0)
2	87/299	85/168	40/198	39/226	1/320	3/285	0/241	75/189	65/199	4/156	5/233	3/340	0/205
	(29.1)	(50.6)	(20.2)	(17.3)	(0.3)	(1.0)	(0)	(39.7)	(32.7)	(2.6)	(2.1)	(0.9)	(0)
3	78/175	72/209	85/290	37/174	3/233	12/224	0/199	82/181	25/169	15/218	56/234	0/130	0/97
	(44.6)	(34.4)	(29.3)	(21.3)	(1.3)	(5.4)	(0)	(45.3)	(14.8)	(6.9)	(23.9)	(0)	(0)
4	40/105	99/186	57/200	27/196	1/157	2/82	0/128	60/168	24/94	32/261	3/95	0/185	0/62
	(38.1)	(53.2)	(28.5)	(13.8)	(0.6)	(2.4)	(0)	(35.7)	(8.2)	(12.3)	(3.2)	(0)	(0)
5	6/42	26/48	5/43	15/74	0/77	2/44	0/33	15/89	4/65	15/116	22/144	0/70	0/42
	(14.3)	(54.2)	(11.6)	(20.3)	(0)	(4.5)	(0)	(16.8)	(6.1)	(12.9)	(15.3)	(0)	(0)
6	14/76	12/37	1/50	22/89	0/56	2/62	0/37	1/64	7/79	0/68	0/50	1/92	0/65
	(18.4)	(32.4)	(2)	(24.7)	(0)	(3.2)	(0)	(1.5)	(8.9)	(0)	(0)	(1.1)	(0)
7	5/43	2/18	1/56	14/64	0/48	1/52	0/53	4/52	0/24	0/33	0/50	0/53	0/37
	(11.6)	(11.1)	(1.8)	(21.9)	(0)	(1.9)	(0)	(7.7)	(0)	(0)	(0)	(0)	(0)
8	4/42	3/12	0/55	8/53	0/46	1/36	0/31	3/54	0/31	0/44	0/35	0/42	0/37
	(9.5)	(25)	(0)	(15.1)	(0)	(2.8)	(0)	(5.5)	(0)	(0)	(0)	(0)	(0)
A	160/358	96/320	102/344	90/322	32/308	0/412	0/174	112/243	112/353	1/447	0/432	0/161	3/455
	(44.7)	(30)	(29.6)	(27.9)	(10.4)	(0)	(0)	(46.1)	(31.7)	(0.2)	(0)	(0)	(0.7)
B	68/264	98/383	163/439	112/288	8/263	2/242	1/282	150/386	210/436	4/365	5/250	0/298	52/451
	(25.7)	(25.6)	(37.1)	(38.9)	(3.0)	(0.8)	(0.3)	(38.9)	(48.2)	(1.1)	(2)	(0)	(11.5)
C	1/24	0/19	0/16	9/42	0/14	0/36	0/37	0/26	0/31	0/19	0/16	0/15	0/23
	(4.2)	(0)	(0)	(21.4)	(0)	(0)	(0)	(0)	(0)	(0)	(0)	(0)	(0)

Data are shown as the median number of positive cells for each marker/median number of total mononuclear cell count in 10 high power field (×400) for each marker for each patient. Percentages are shown in brackets. Chronic Chagas disease patients with advanced heart disease (cChHD) [1]–[8]; Patients suffering from Giant Cell Myocarditis (GCM) [A and B]; Patient with Idiopathic Dilated Cardiomyopathy (IDCM) [C].

### Analysis of cardiac explants specimens

**Figure 1:**
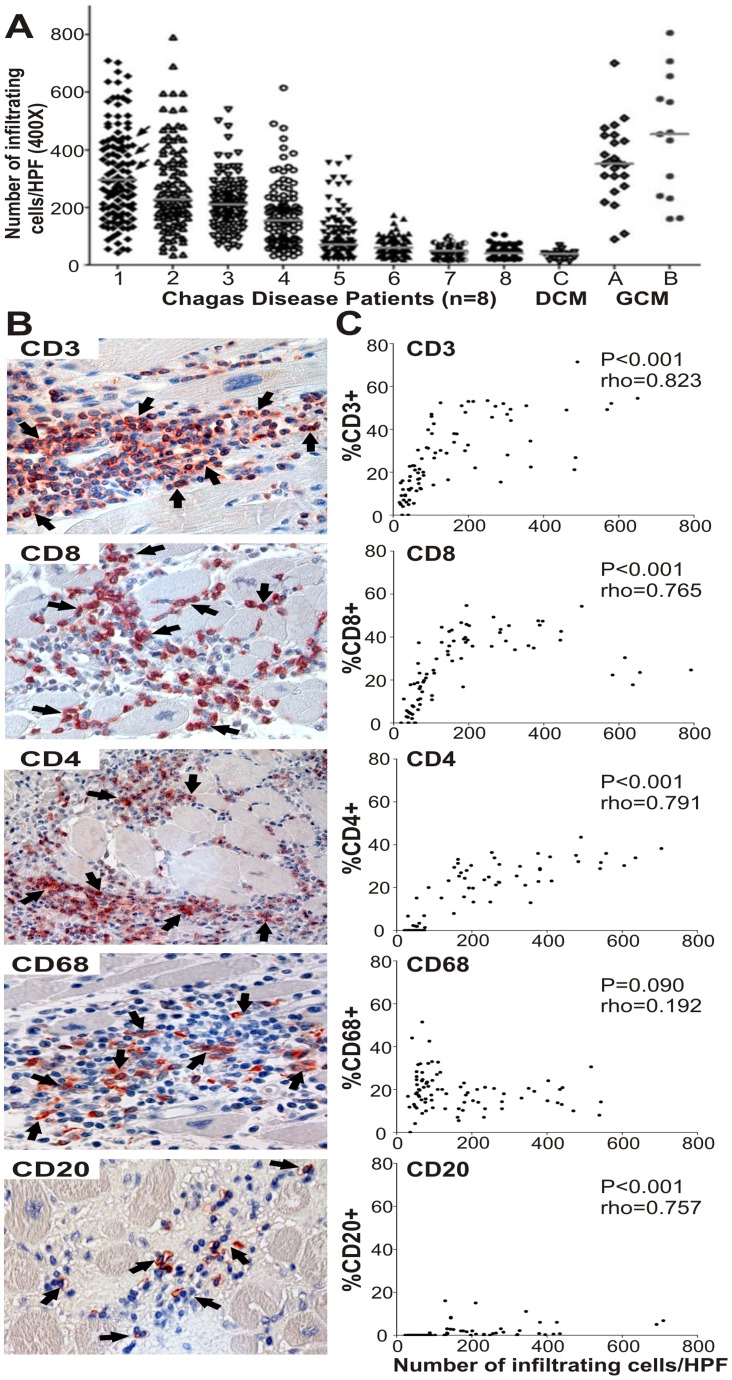
Degree of inflammation and cell type composition of inflammatory cell infiltrates in heart samples from patients with chronic Chagas disease. A) The total number of mononuclear inflammatory cells were counted in 10 HPF [Original Magnification (O.M.) 400×] from the interventricular septum of 13 sections assessed for each patient with Chagas disease (n = 8), GCM (n = 2) or DCM (n = 1). Each dot represents the total number of mononuclear inflammatory cells in one out of the 130 HPFs counted for each patient. The horizontal lines indicate median values. Arrows indicate the HPF in which parasites were detected by direct observation. B) Representative photos of immunohistochemistry showing total T cells (CD3^+^), cytotoxic T cells (CD8^+^), helper T cells (CD4^+^), macrophages/dendritic cells (CD68^+^) and B cells (CD20^+^). Arrows show positive staining for the corresponding marker. C) Correlation analysis between the percentage of cells expressing each marker and the total number of mononuclear inflammatory cells in chronic Chagas disease patients (n = 8) was performed by Spearman correlation test.

The mean weight of cChHD explanted hearts was 450±65 g. Four∶8 cChHD interventricular septum samples showed severe diffuse myocarditis, 2∶8 moderate myocarditis and 2∶8 had mild focal myocarditis. Interstitial fibrosis was moderate to severe in all except for one interventricular septum sample. Amastigote nests were only recorded in 3 sections with severe diffuse myocarditis of patient 1 (Table 1, Figure 1A). However, the presence of *T. cruzi* by PCR in the interventricular septum of patients 1–5 and 7–8 but not in patient 6 was demonstrated in a previous report [23]. The mean weight of hearts with GCM was 375 g and exhibited severe diffuse myocarditis and severe fibrosis. The IDCM heart weighted 510 g, presenting moderate fibrosis and absence of myocarditis (Table 1).

### Characterization of the inflammatory cell infiltrate in the hearts of patients with severe chronic Chagas disease

Inflammatory mononuclear cell infiltrates in heart tissue samples from cChHD were evaluated by immunohistochemistry- As observed in Figure 1A and Table 2, patients 1 to 4 exhibited severe diffuse inflammatory infiltrate, while patients 5 to 8 presented lower number of total infiltrating cells within the interventricular septum. The percentage of T (CD3^+^, CD4^+^, CD8^+^) and B cells (CD20^+^) was correlated with the total cell count in inflammatory cell infiltrates (Figure 1B and 1C). Conversely, the percentage of macrophages/dendritic cells (CD68^+^ cells) in regions with the highest levels of infiltration was similar to those observed in areas with low inflammation (Figure 1B and 1C). In 7 out of the 8 cChHD, CD8^+^ T cells were prevalent among infiltrating mononuclear cells compared to CD4^+^, CD68^+^ and CD20^+^ cells (Table 2).

### T cell differentiation of infiltrating cells in the heart of patients with Chagas disease myocarditis

**Figure 2:**
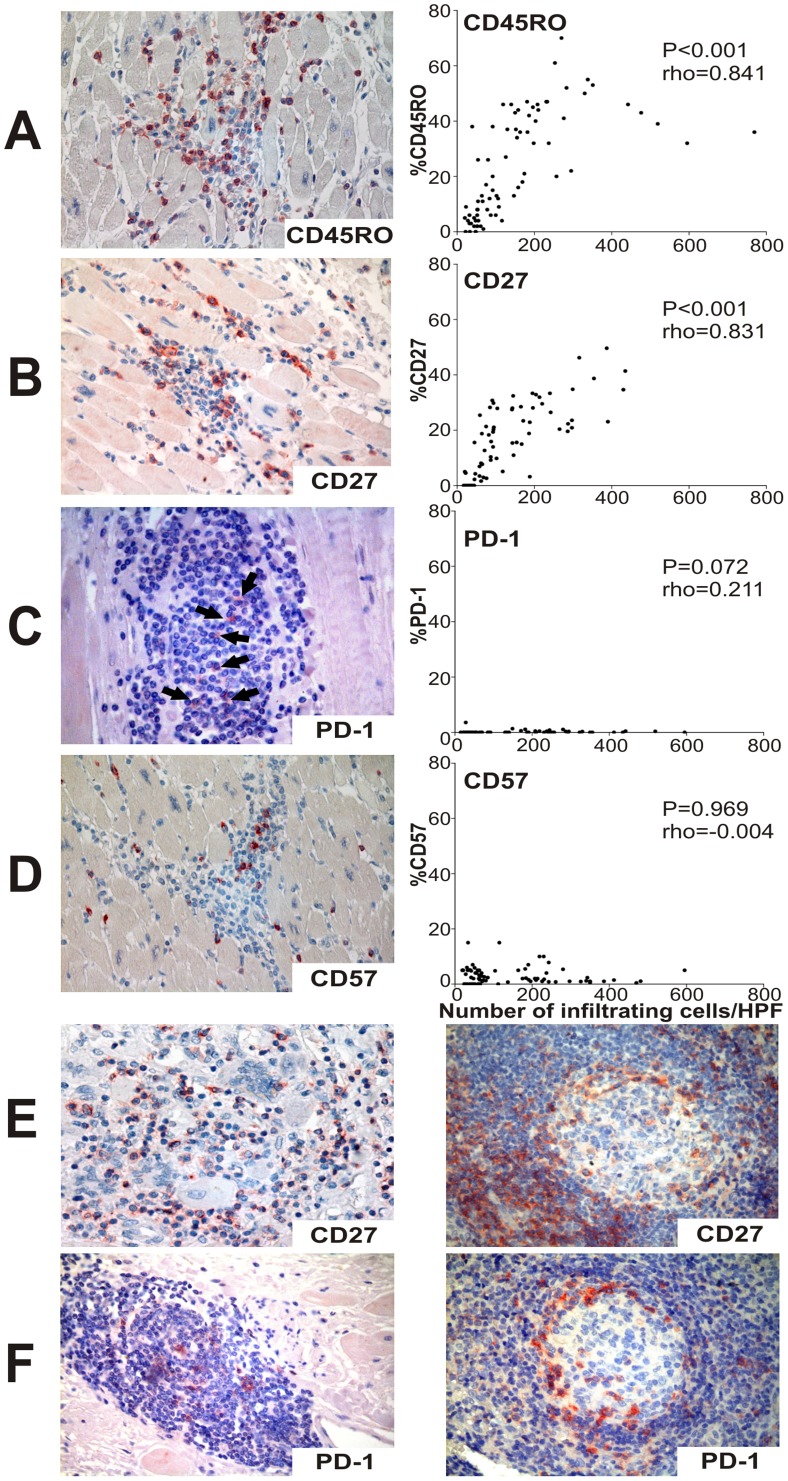
Expression of markers of T cell differentiation and degree of inflammation in the heart of chronically *T. cruzi*-infected subjects with severe cardiomyopathy. A–D, left panel: representative photos of CD45RO, CD27, PD-1 and CD57 expression, respectively. A–D, right panel. Each dot represents the percentage of cells expressing each marker (Y axis) vs. the total number of mononuclear inflammatory cells (X axis) in each HPF out 10 HPF (O.M. 400×) evaluated for patient (n = 8). Correlation analysis was done by Spearman correlation test. Arrows in C indicate positive staining for PD-1. E) CD27 expression in the heart from one GCM patient (left panel) and in control lymph node tissues (right panel). F) PD-1 expression in myocardial lymphoid follicles (tertiary lymphoid follicles) in the heart of one chagasic patient (left panel) and in control lymph node tissues (right panel).

Since T cells were the prevalent cell population in heart tissues with high degree of inflammation, the expression of markers of antigen-experienced T cells and T cell differentiation (Table S1) was evaluated in heart samples. A high percentage (>30%) of infiltrating cells in heart tissues from cChHD with severe myocarditis express CD45RO, a marker of antigen-experienced T cells (Figure 2A, patients 1–4 in Table 2). Likewise, CD27 expression, a marker of low grade T cell differentiation was higher in cChHD with severe myocarditis than in patients with lower degree of inflammation (Figure 2B, patients 1–4 vs. patients 5–8 in Table 2). In contrast, the expression of the inhibitory receptor PD-1, the immunosenescence marker CD57 and CD45RA (i.e. expressed by naïve and terminally differentiated T cells; Table S1), was generally very low in heart tissues from cChHD (Figure 2C, 2D, Figure S1, Table 2). Since CD57 is expressed in terminally differentiated effector T cells and also in NK, the low expression of CD57 confirms the scarcity of NK in heart tissue samples from chronic Chagas disease patients [3]. PD-1 expression was detected in intramyocardial lymphoid follicles (tertiary lymphoid follicles) in the heart of patients with severe Chagas myocarditis (i.e. patients 1–4) [Figure 2C and 2F, left panel]. Of note, tertiary lymphoid follicles were randomly observed in myocardial tissues, as confirmed by the expression of CD21 and PD-1. These findings indicate that antigen-experienced T cells with low grade of differentiation are abundant in Chagas disease myocarditis.

### Infected cardiac myocytes are surrounded by cells bearing memory markers

To better characterize the phenotype of the cell infiltrate recruited by infected myocytes, tissue samples bearing amastigotes nests along with high inflammation were selected and analyzed for the expression of CD45RO, CD45RA, CD57, CD68 and CD20 (Figure S1). Most cells surrounding the infected cardiomyocyte were CD45RO^+^, CD45RA^−^, CD57^−^, CD20^−^; while a lower number expressed CD68^+^ (Figure S1A and S1B).

With the aim to evaluate whether the different cell types were recruited with the same efficacy towards the infected cardiomyocyte, the spatial pattern of CD45RO^+^ and CD45RO^−^ cells was analyzed by using the O-ring test for 2-dimensional point patterns. A statistically significant aggregation of CD45RO^+^ cells (blue line in Figure S1D) was observed near the infected cardiomyocyte (i.e. CD45RO^+^ cells are over the confidence envelope) [Figure S1C and S1D, P<0.001]. The proportion of CD45RO^+^ cells decreases at longer distances from the infected cell.

### Functional profile of T cells in Chagas disease myocarditis: High number of T-bet- and Ki67-expressing lymphocytes and low number of FOXP3^+^ cells

**Figure 3:**
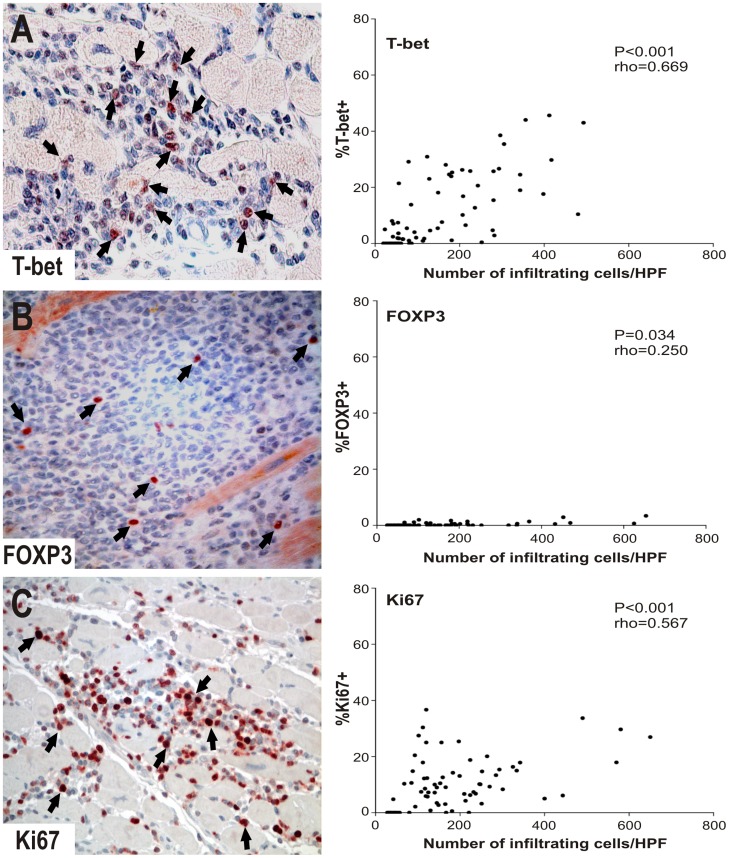
T-bet, FOXP3 and Ki67 expression in cChHD myocarditis. A) Representative photo (O.M. 400×) of T-bet^+^ cells (arrows) in cChHD, (left panel). Percentage of T-bet^+^ cells vs. total mononuclear inflammatory cells counted in 10 representative HPFs for each cChHD (n = 8), [right panel]. B) Representative photo (O.M. 400×) of FOXP3 in cChHD, left panel. Arrows indicate FOXP3^+^ cells. Percentage of FOXP3^+^ cells vs. total mononuclear inflammatory cells counted in 10 representative HPFs for each cChHD (n = 8), right panel. C) Representative photo (O.M. 400×) of Ki67 in cChHD, left panel. Arrows indicate Ki67^+^ lymphocytes in cChHD. Percentage of Ki67^+^ cells vs. total mononuclear inflammatory cells counted in 10 representative HPFs for each cChHD (n = 8), right panel. Correlation analysis for all markers was carried out by Spearman correlation test.

**Figure 4:**
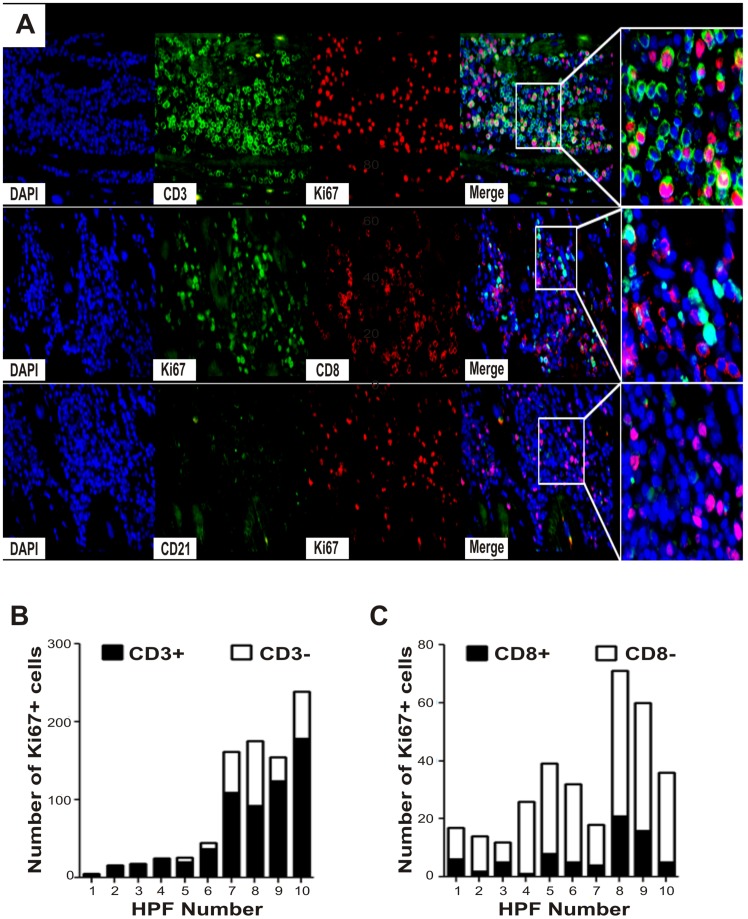
Proliferation of T and B cells in Chagas disease myocarditis. A) Representative photos (O.M. 400×) of double immunofluorescence staining of Ki67 with CD3 (upper row); CD8 (middle row); or CD21 (lower row) cell. All images correspond to patient 1. Nuclei were stained with DAPI (blue). Single CD3 (green), single Ki67 (red) and double CD3 and Ki67 staining (merge) are shown in the upper row (inset, arrows). Single CD8 (red), single Ki67 (green) and double CD8 and Ki67 staining are shown in the middle row (inset, arrow). Single CD21 (green) and single Ki67 (red) expression is shown in the lower row (inset, arrow). Proportion of total Ki67^+^ cells showing CD3 (B) or CD8 (C) expression (filled columns), in 10 HPFs (numbered from 1 to 10) of patient 1.

The functional profile of the cell infiltrate was assessed by the expression of T-bet, a marker of type 1 T cell responses, the regulatory molecules FOXP3 and HLA-G, and Ki67, a marker of proliferative potential (Table S1). T-bet expression was broadly detected in cell nuclei in areas of high cell infiltrate and was correlated with the degree of cell infiltration (P<0.001, rho = 0.669) (Figure 3A; Table 2). In contrast, HLA-G^+^ and FOXP3^+^ cells were found in a very low proportion or were absent in cChHD (Figure 3B, Table 2). Cells with proliferative potential were observed in tissue samples from patients with severe myocarditis (median percentage of proliferating cells = 9%) in areas with high inflammation (Figure 3C, Table 2 and Table 3). In order to identify the proliferating lymphocyte subsets, double immunofluorescence staining was carried out using Ki67, CD3, CD8 and CD21 antibodies (Figure 4). Most cells expressing Ki67^+^ were CD3^+^ cells [median percentage (interquartile range) = 74 (66–89)], while a minor proportion of Ki67^+^ cells expressed CD8 [median percentage (interquartile range) = 21 (15–19)], (Figure 4A, 4B and 4C). In contrast, a very low proportion of Ki67^+^ cells expressed CD21 (data not shown), suggesting that KI67^+^/CD21^−^ proliferating cells belong to the T cell compartment.

**Table 3:** Comparative analysis between inflammatory cell infiltrates of cChHD and GCM.

Markers	cChHDsM (n = 4)	GCM(n = 2)	P value cChHDsM vs. GCM
Cell type			
CD3	87/206 (42.2)	137/330 (41,5)	0.374
CD8	92/205 (44.9)	97/269 (36.1)	0.483
CD4	76/272 (27.9)	132/392 (33.7)	0.009
CD68	36/224 (16.1)	101/312 (32.4)	<0.001
CD20	3/211 (1.4)	7/275 (2.5)	0.147
Differentiation			
CD57	3/222 (1.3)	2/313 (0.6)	0.001
PD-1	0/231 (0)	1/229 (0.4)	0.146
CD45RO	82/203 (40.4)	120/355 (33.8)	0.541
CD27	45/187 (24.1)	154/425 (36.2)	<0.001
Functional			
Ki67	16/222 (7.2)	1/408 (0.2)	<0.001
T-bet	30/206 (14.6)	1/426 (0.2)	<0.001
FOXP3	0/182 (0)	0/261 (0)	0.092
HLA-G	0/137 (0)	24/455 (5.3)	<0.001

Data are shown as the median number of positive cells for each marker/median number of total mononuclear cell count in 10 HPF (400×) for each marker. Percentages are shown in brackets.

Significant differences between groups were assessed by Mann- Whitney *U* test. cChHDsM, chronic Chagas heart disease with severe myocarditis (patients 1–4 of Table 3); GCM, Giant Cell Myocarditis; HPF, high power field.

### Comparison of phenotypic and functional profiles between chronic Chagas disease myocarditis and Giant Cell Myocarditis

To assess whether the phenotypic and functional profiles found in Chagas disease myocarditis were associated with parasite persistence, the same set of markers were assessed in heart tissues from patients suffering from idiopathic GCM (patient A and B, Table 2), and compared to the profile in cChHD with severe myocarditis (patients 1–4, Table 2). GCM is characterized by an intense myocarditis with multifocal cardiomyocyte damage. Although T lymphocytes were a major cell type in both severe cChHD and GCM, the latter was enriched in macrophages/dendritic cells and CD4^+^ T cells (Table 3), whereas CD8 was prevalent in cChHD with severe myocarditis.

Regarding the differentiation status of the cell infiltrate in these two types of myocarditis, no differences were found in PD-1 and CD45RO expression, whereas CD57 expression was low in both types of myocarditis. cChHD with severe myocarditis presented lower levels of CD27^+^ cells (i.e. 2/4 patients with severe myocarditis in Table 2) compared to GCM (Table 2 and Table 3). Higher counts of Ki67^+^ and T-bet^+^ cells were found in cChHD as compared with GCM, whereas the expression of FOXP3 was very low in both myocarditis. HLA-G expression was only recorded in cardiomyocytes and mononuclear inflammatory cells from GCM (Table 2 and Table 3). Altogether, these findings support the notion that chronic infection with *T. cruzi* drives T cell differentiation and proliferation in chronic Chagas disease myocarditis.

## Discussion

Most studies concerning the characterization of inflammatory cells in heart tissues in chronic Chagas disease have comprised the evaluation of different cell types, and the cytokine profile, while the differentiation status of T cells is less known. Herein, we report that antigen-experienced (CD45RO^+^) T cells with a low degree of differentiation (CD27^+^/CD57^−^), a Th1 profile (Tbet^+^) and proliferative capacity (Ki67^+^) are recruited into the heart of patients with chronic advanced Chagas disease myocarditis. As previously described, the inflammatory cell infiltrate in most patients was dominated by CD8^+^ T cells [2], [3], [6]. Although less prominent than CD8^+^ T cells, appreciable counts of CD4^+^ T cells were also found in intracardiac infiltrates.

The T cell phenotype in heart tissues concurs with that observed in circulating CD8^+^ and CD4^+^ T cells responsive to *T. cruzi* antigens in patients with chronic Chagas disease, regardless the clinical status [15], [16], but it is at odds with the phenotype of total peripheral CD4^+^ and CD8^+^ T cells [15]–[17], [Table S2, [8], [10]–[13], [15]–[17], [40]–[43]. Whereas most circulating IFN-γ-producing CD8^+^ and CD4^+^ T cells responsive to *T. cruzi* display a low degree of differentiation (CD27^+^/CD28^+^/CD57^−^/LIR-1^−^) [8], [16], high differentiated (CD27^−^/CD28^−^/CD57^+^/LIR-1^+^) CD8^+^ and CD4^+^ T cells are increased in the total peripheral T cell compartment of patients with severe cardiomyopathy [8], [15]–[17]. Subjects with chronic *T. cruzi* infection with severe cardiomyopathy also displayed lower frequencies of *T. cruzi*-responsive IFN-γ-producing T cells and lower levels of IFN-γ production compared to subjects with no signs of heart disease [8], [13], [16], leading us to propose that long-term parasite persistent might dampen parasite specific T cell responses [44]. Thus, recently developed effector T cells that bear a low degree of differentiation appear to be an important source of *T. cruzi*-specific T cells in the periphery of chronic Chagas disease patients. In contrast, the bulk of the total CD8^+^ and CD4^+^ T cells might reflect the effect of persistent exposure to the parasite.

The low degree of differentiation of T cells found in the heart of chronically *T. cruzi*-infected subjects was somehow unexpected, since T cells at target tissues are more likely to be stimulated by antigen and induced to further differentiation. However, CD27^−^ T cells also comprised a substantial fraction of the inflammatory infiltrate, indicating a heterogeneous composition of T cells with different degree of differentiation in heart tissues. The presence of CD27^+^/CD57^−^ cells might account for proliferating CD3^+^ T cells, as proliferation is a hallmark of T cells with low grade of differentiation (CD27^+^ cells) and low rounds of antigen stimulation (CD57^−^ T cells) [45].

We also demonstrate that antigen-experienced (CD45RO^+^) T cells were more efficiently recruited than other mononuclear cells towards the infected cardiomyocyte, supporting that *T. cruzi* is a driving force for T cell recruitment. Activated VLA-4^+^/LFA-1^+/^granzyme A^+^ T lymphocytes have been observed in cardiac infiltrates of patients with chagasic heart failure [7], while the local production of IL-7 and IL-15 was claimed to be associated with the maintenance and predominance of CD8^+^ T cells in heart tissues [46]. However, the recruitment of early effector T cells might be also involved in the maintenance of inflammatory cells at sites of chronic localized infection.

Tertiary lymphoid organs associated to severe myocarditis might have been developed in the context of chronic inflammatory conditions [47]–[49]. Since the formation of TLO involves the recruitment of lymphocytes not normally associated with inflammatory infiltrates, notably naive T cells and central memory T cells, these structures might be a source of recently recruited effector T cells from the naïve or central memory pool. To the best of our knowledge, there are not reports describing these structures in chronic chagasic cardiomyopathy. TNF-α has been pointed out as a key molecule in the generation of ectopic TLO which might facilitate the perpetuation of inflammation in the heart. Cells expressing TNF-α and IFN-γ have been identified in heart tissues from patients with chronic chagasic cardiomyopathy [9], [50]. Our findings showing T-bet expression in areas of high inflammation and proliferation further confirmed a type 1 T cell response in patients with severe heart disease. The low levels of PD-1 counts concur with the presence of T-bet^+^ cells, since T bet inhibits PD-1 expression [51].

Recent reports have demonstrated that regulatory T cells (FOXP3^+^) are absent in Chagas disease myocarditis, indicating that deficiency in IL-10 producing T regs may led to a regulatory imbalance, perhaps rendering heart tissues increasingly susceptible to type 1-dependent pathology [9], [52]. In line with these findings, scarce FOXP3^+^ cells were found in heart tissues from patients with severe heart disease. Nevertheless, we have shown that CD3^+^ T lymphocytes infiltrating heart tissues express CTLA-4, another negative regulator of effector T cell responses [8]. In addition to be involved in the regulatory T cell function of T regs, CTLA-4 is also upregulated on activated T cells [53]. This might explain the concomitant presence of proliferating and CTLA-4^+^ T cells. However, it is likely that proliferating T lymphocytes do not CTLA-4 or likely express dysfunctional CTLA-4.

The predominant role for *T. cruzi* in the differentiation and functional T cell profile observed in the heart of subjects with chronic *T. cruzi* infection becomes apparent at comparing the inflammatory cell infiltrates between chagasic and GCM myocarditis which is a rare and frequently fatal type of myocarditis with features of autoimmune disease [54], [55]. Higher levels of infiltrating cells with low degree of differentiation (i.e. CD27^−^ expressing cells), lower T-bet expression and lower proliferative capacity were observed in GCM compared with severe Chagas disease myocarditis. Of note, HLA-G, a non-classical class I major histocompatibility complex molecule playing a tolerogenic role in innate and adaptive responses [56] was not expressed in inflammatory cell infiltrates of chronic Chagas disease patients, while it was expressed by cardiomyocytes in GCM. These findings support that immunoregulation is different between an infectious and a non-infectious myocarditis. Coxsackievirus infection is another common cause of infectious myocarditis, in which a pathogen driven inflammatory process was supported by the reduction in inflammation and resolution of the infection after treatment with IFN-beta [57], [58].

The development of tissue damage might depend on parasite burden; the effectiveness of the host immune response in controlling parasite replication, and the effectiveness of the host immune response in limiting peripheral damage. The effectiveness of the host immune response in controlling parasite replication without the induction of tissue damage will largely rely on the quality of T cell responses [59]. We have recently reported that children in early stages of *T. cruzi* infection maintain polyfunctional and stronger T cell responses to *T. cruzi* in their circulation, contrasting with the prevalent monofunctional T cell profile in long-term *T. cruzi*-infected adults [60]. Although, we cannot rule out that recruitment of IFN-γ-producing T cells into the heart might account for the decrease of IFN-γ-producing T cells from the periphery, it is also possible that clonal exhaustion occurs overtime. Consequently, in the phase of an ineffective T cell response, the development of tissue damage might occur in order to get parasites under control. Similarly, immunoregulatory pathways might be also dampened overtime.

In summary the quality of T cell responses and immunoregulatory mechanisms might determine the pattern of the cellular response and the severity of disease in chronic *T. cruzi* infection.

### Limitation of the study

The main limitation of the study is the sample size. However, the detailed analysis conducted and the difficulty to get tissue samples can mitigate, at least partially, such limitation.

## Supporting Information

Figure S1 Spatial analysis of infiltrating cells surrounding *T. cruzi*-infected cardiomyocyte in heart tissues from patients with chronic Chagas disease. A) Immunohistochemistry staining of the infected cardiomyocyte showing CD45RO, CD45RA, CD57 and CD68 expression [O.M. 400×]. Open arrows show amastigote nests while black arrows indicate positive staining for the corresponding marker. B) Immunohistochemistry assay [O.M. 100×] showing antigen-experienced CD45RO^+^T cells surrounding the infected cardiomyocyte. C) Spatial analysis of CD45R0^+^ T cells surrounding an infected human cardiomyocyte. Cell positioning dot plot showing 933 CD45RO^+^ (red dots) and 428 CD45RO^−^ (blue dots) T cells. D) Statistics of the spatial distribution of CD45RO^+^ cells at different distances (r) from the infected cardiomyocyte was performed using the O-ring test [24]–[25]. The statistic values are depicted as O12 (r) (blue line). The confidence envelopes (P<0.001, grey lines, E12^+^ and E12^−^) were determined by 999 Monte Carlo simulations. The upper limit of the confidence envelope (upper grey line, E12^+^) delimits statistically significantly aggregation of CD45RO^+^ cells; while the lower limit (E12^−^) delimits statistically significant repulsion of these cells. Values between E12^+^ and E12^−^ do not differ significantly from randomness.
(TIF)

Table S1 Note: * Unless otherwise stated, antibodies were unconjugated mouse monoclonal antibodies. † CD8a is also expressed in some dendritic cell subsets, and can be induced in NKs and HDE CD4 T cells. ‡ CD4 is expressed by monocytes and can be induced in macrophages and dendritic cells. DE, Terminally Differentiated Effector T cells; TCM, Central Memory T cells; TEM, Effector Memory T cells.
(DOCX)

Table S2 ^A^ *T. cruzi*-infected vs. uninfected controls. ^B^ PBMC were stimulated with *T. cruzi* antigens. E, effector; CM, central memory; EM, effector memory; TET, terminally differentiated effector; cChHD, advanced chronic Chagas heart disease.
(DOCX)
