# Supplementary figures and images for: Presence of Antigen-Experienced T Cells with Low Grade of Differentiation and Proliferative Potential in Chronic Chagas Disease Myocarditis
Source: PLoS Negl Trop Dis. 2014 Aug 21;8(8):e2989. doi: 10.1371/journal.pntd.0002989 (PMC4140664; doi:10.1371/journal.pntd.0002989)

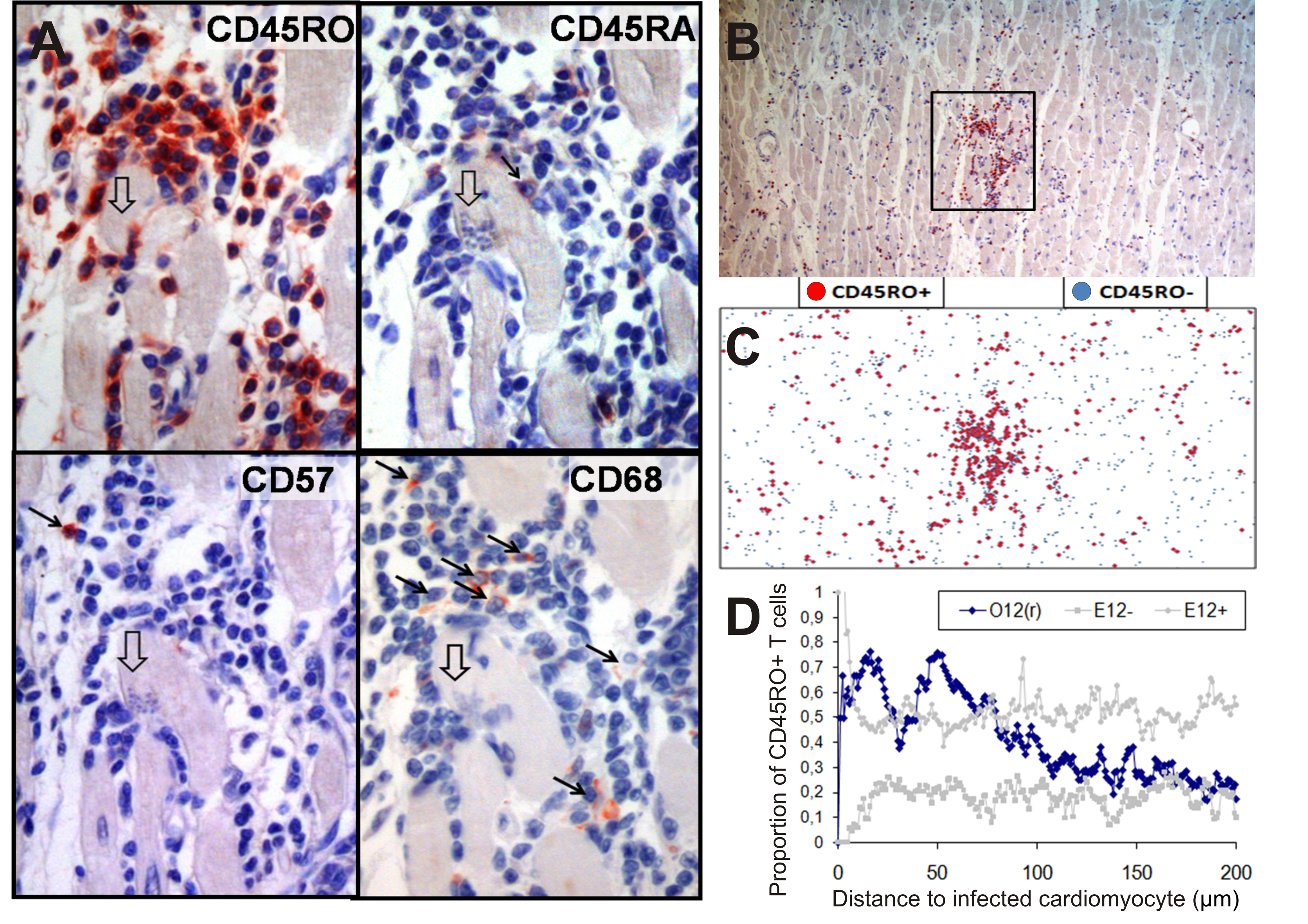

Supplement: Figure S1 — Spatial analysis of infiltrating cells surrounding T. cruzi-infected cardiomyocyte in heart tissues from patients with chronic Chagas disease. A) Immunohistochemistry staining of the infected cardiomyocyte showing CD45RO, CD45RA, CD57 and CD68 expression [O.M. 400×]. Open arrows show amastigote nests while black arrows indicate positive staining for the corresponding marker. B) Immunohistochemistry assay [O.M. 100×] showing antigen-experienced CD45RO+T cells surrounding the infected cardiomyocyte. C) Spatial analysis of CD45R0+ T cells surrounding an infected human cardiomyocyte. Cell positioning dot plot showing 933 CD45RO+ (red dots) and 428 CD45RO− (blue dots) T cells. D) Statistics of the spatial distribution of CD45RO+ cells at different distances (r) from the infected cardiomyocyte was performed using the O-ring test [24]–[25]. The statistic values are depicted as O12 (r) (blue line). The confidence envelopes (P<0.001, grey lines, E12+ and E12−) were determined by 999 Monte Carlo simulations. The upper limit of the confidence envelope (upper grey line, E12+) delimits statistically significantly aggregation of CD45RO+ cells; while the lower limit (E12−) delimits statistically significant repulsion of these cells. Values between E12+ and E12− do not differ significantly from randomness. (TIF) [file pntd.0002989.s001.tif]
