# Supplementary material for: Presence of Antigen-Experienced T Cells with Low Grade of Differentiation and Proliferative Potential in Chronic Chagas Disease Myocarditis
Source: PLoS Negl Trop Dis. 2014 Aug 21;8(8):e2989. doi: 10.1371/journal.pntd.0002989 (PMC4140664; doi:10.1371/journal.pntd.0002989)
Supplement: Table S1 — Note: * Unless otherwise stated, antibodies were unconjugated mouse monoclonal antibodies. † CD8a is also expressed in some dendritic cell subsets, and can be induced in NKs and HDE CD4 T cells. ‡ CD4 is expressed by monocytes and can be induced in macrophages and dendritic cells. DE, Terminally Differentiated Effector T cells; TCM, Central Memory T cells; TEM, Effector Memory T cells. (DOCX) [file pntd.0002989.s002.docx]

Table S1. Principal features of study molecules.

| **Marker** | | **Antibody Clone *** | **Cellular expression** | **Function** | |
| --- | --- | --- | --- | --- | --- |
| Cell type | **CD3** | Rabbit polyclonal | T cells | TCR signaling^24^ |  |
|  | **CD8a** | 1A5 | Cytotoxic T cells † | Class I restriction of TCR^25^ |  |
|  | **CD4** | 1F6 | Helper T cells ‡ | Class II restriction of TCR^25^ |  |
|  | **CD68** | KP1 | Macrophage and dendritic cells | Lysosomal granules of phagocytic cells^26^ |  |
|  | **CD20** | L26 | B cells | BCR signaling^27^ |  |
|  | **CD21** | EP3093, Rabbit mAb | Follicular dendritic cells and B cells | Complement receptor 2^28^ |  |
| Differentiation | **CD57** | HNK-1 | TDE and NK cells | Nº TCR events, effector function, senescence^29^ |  |
|  | **PD-1** | NAT | TDE and B cells | Inhibitory receptor of follicular helper T cells and TDE cells^30,31^ |  |
|  | **CD45RA** | MT2 | T naïve, TDE and B cells | Activation signaling^32^ |  |
|  | **CD45RO** | UCHL-1 | TCM and TEM | Ag experience – Signalling^32^ |  |
|  | **CD27** | 137B4 | T naïve, TCM, TEM and plasma cells | Co-stimulation^33^ |  |
| Functional | **T-bet** | 4B10 | TH1 T cells | Type 1 response. Induced by IFN-γ through STAT-1 on T and B cells. Potentiates IL-12 responsiveness^34^ |  |
|  | **FOXP3** | 259D/C7 | Regulatory T cells | Regulates constitutive CD25 and CTLA-4 expression by Tregs^35^ |  |
|  | **HLA-G** | 4H84 | Macrophages and muscle cells | Ligand of LIR-1 and KIR2DL4^36^ on T cells. |  |
|  | **Ki67** | MM1 EPR3610, Rabbit mAb | All cells in active cell cycle | Proliferation (Expressed in G1, S, G2 and M) ^37^ |  |
