# Supplementary material for: Presence of Antigen-Experienced T Cells with Low Grade of Differentiation and Proliferative Potential in Chronic Chagas Disease Myocarditis
Source: PLoS Negl Trop Dis. 2014 Aug 21;8(8):e2989. doi: 10.1371/journal.pntd.0002989 (PMC4140664; doi:10.1371/journal.pntd.0002989)
Supplement: Table S2 — A T. cruzi-infected vs. uninfected controls. B PBMC were stimulated with T. cruzi antigens. E, effector; CM, central memory; EM, effector memory; TET, terminally differentiated effector; cChHD, advanced chronic Chagas heart disease. (DOCX) [file pntd.0002989.s003.docx]

Table S2. Phenotype of total and *T.cruzi*-responsive T cells and IFN-γ-production in the periphery of

long-term chronically *T. cruzi*-infected subjects.

| **T cell phenotype (%)** | **Cell expression** | **Findings** | **References** |
| --- | --- | --- | --- |
|  | **on T cells** |  |  |
| *Gated on total lymphocytes* |  |  |  |
| CD8^+^ |  | Normal ^A^ | 8,15,41 |
| CD8^+^CD45RA^+^ | Naïve, TET | Decreased ^A^ | 40 |
| CD8^+^CD28^+^ | Naïve, CM | Decreased ^A^ | 17,42 |
| CD8^+^CD45RA^—^CD27^+^CD28^+^ | CM | Decreased ^A^ | 15 |
| CD8^+^LIR-1^+^ | EM, TET | cChHD>asymptomatic> | 8 |
|  |  | uninfected |  |
| CD4^+^ |  | Normal ^A^ | 8,16,41 |
| CD4^+^CD45RA^+^ | Naïve, TET | Decreased ^A^ | 40 |
| CD4^+^CD45RA^+^CD27^+^CD28^+^ | Naive | Decreased  ^A^ | 16 |
| CD4^+^CD45RA^+^CD27^—^CD28^—^CD57^+^ | TET | Increased ^A^ | 16 |
| CD4^+^ LIR-1^+^ | TET | Increased ^A^ | 8 |
|  |  |  |  |
| *Gated on CD8^+^IFN-γ^+^ T cells responsive to T. cruzi*. ^B^ | E |  |  |
| CD27^+^CD28^+^ |  | cChHD = asymptomatic | 15 |
|  |  |  |  |
| *Gated on CD4^+^IFN-γ^+^ T cells responsive to T. cruzi*. ^B^ | E |  |  |
| CD27^+^CD28^+^ |  | cChHD = asymptomatic | 16 |
|  |  |  |  |
| **Peripheral IFN- γ production in response to *T. cruzi*** ***^B^*** |  |  |  |
| Levels of IFN-γ | E | cChHD > asymptomatic | 10, 11 |
|  |  | cChHD < asymptomatic | 8 |
| Number of IFN**-γ-**producing cells | E | cChHD < asymptomatic | 13 |
| % CD8^+^IFN- γ^+^ | E | cChHD < asymptomatic | 15 |
|  |  | cChHD < asymptomatic | 43 |
|  |  | cChHD > asymptomatic | 12 |
| % CD4^+^IFN- γ^+^ | E | cChHD > asymptomatic | 11 |
|  |  | cChHD < asymptomatic | 16 |
|  |  | cChHD = asymptomatic | 12 |
| %CD4+CD45RO^+^IFN-γ^+^ | E | cChHD = asymptomatic | 40 |
| IFN-γ mRNA from PBMC | E | cChHD < asymptomatic | 14 |
